# Supplementary material for: Expert opinion as priors for random effects in Bayesian prediction models: Subclinical ketosis in dairy cows as an example
Source: PLoS One. 2021 Jan 14;16(1):e0244752. doi: 10.1371/journal.pone.0244752 (PMC7808599; doi:10.1371/journal.pone.0244752)
Supplement: S2 Appendix — (DOCX) [file pone.0244752.s002.docx]

**Appendix B**

R code for Bayesian prediction model without incorporation of expert prior knowledge

Three steps:

1. Use ‘rstan’ package to get posterior estimates for the parameters
2. Save the posterior iterations from all chains per parameter
3. Predict the SCK risk per animal

## Step 1: use ‘rstan’ to get the posterior estimates for the parameters ##

## First specify the model and the priors in “model development.stan”

data {

int<lower=0> Nk;

int<lower=0> Nj;

int<lower=0,upper=1> y[Nk];

int<lower=1> herds[Nk];

vector[Nk] parity2;

vector[Nk] parity3;

vector[Nk] parity4;

vector[Nj] spring;

vector[Nj] winter;

vector[Nj] summer;

vector[Nk] bhbz_f;

vector[Nk] acet_f;

vector[Nk] ve_rat;

}

parameters {

real beta_0;

real beta_1;

real beta_2;

real beta_3;

real beta_4;

real beta_5;

real beta_6;

real beta_7;

real beta_8;

real beta_9;

// Level-2 random effect

real u_j[Nj];

real sigma_u;

}

transformed parameters {

real herd_level[Nj];

real mu_herd[Nk];

for (j in 1:Nj) {

herd_level[j] <- beta_4 * spring[j] + beta_5 * winter[j] + beta_6 * summer[j] + u_j[j];

}

for (i in 1:Nk) {

mu_herd[i] <- herd_level[herds[i]];

}

}

model {

// non-informative priors

beta_0 ~ normal(0,100);

beta_1 ~ normal(0,100);

beta_2 ~ normal(0,100);

beta_3 ~ normal(0,100);

beta_4 ~ normal(0,100);

beta_5 ~ normal(0,100);

beta_6 ~ normal(0,100);

beta_7 ~ normal(0,100);

beta_8 ~ normal(0,100);

beta_9 ~ normal(0,100);

// Random effects u_j

u_j ~ normal(0, sigma_u);

pow(sigma_u, 2) ~ inv_gamma(0.001, 0.001);

// Likelihood

for (i in 1:Nk) {

y[i] ~ bernoulli_logit(beta_0 + beta_1 * parity2[i] + beta_2 * parity3[i] + beta_3 * parity4[i]+ beta_7 * bhbz_f[i] + beta_8 * acet_f[i] + beta_9 * ve_rat[i]+ mu_herd[i]);

}

}

## then run the model with 3 chains, with each chain having 5000 burn-in iterations and the saved 20000 iterations thinned by 100

fileName <- './model development.stan'

stan_code <- readChar(fileName, file.info(fileName)$size)

cat(stan_code)

development <- stan(model_code = stan_code, data = data, chains = 3, iter = 25000, warmup = 5000, thin = 100)

# saved posteriors for parameters

print(development, pars = c('beta_0', 'beta_1', 'beta_2', 'beta_3', 'beta_4', 'beta_5', 'beta_6','beta_7', 'beta_8', 'beta_9','sigma_u'), digits=4)

# traceplots

rstan::traceplot(development, pars = c('beta_0', 'beta_1', 'beta_2', 'beta_3', 'beta_4', 'beta_5', 'beta_6','beta_7', 'beta_8', 'beta_9','sigma_u'), inc_warmup = FALSE)

## Step 2: save the posterior iterations per parameter for prediction ##

stan.output <-extract(development, permuted = TRUE, inc_warmup = FALSE)

post.beta0<- stan.output[[1]]

post.beta1<- stan.output[[2]]

post.beta2<- stan.output[[3]]

post.beta3<- stan.output[[4]]

post.beta4<- stan.output[[5]]

post.beta5<- stan.output[[6]]

post.beta6<- stan.output[[7]]

post.beta7<- stan.output[[8]]

post.beta8<- stan.output[[9]]

post.beta9<- stan.output[[10]]

post.sdU <- stan.output[[12]]

}

## Step 3: predict for each animal the SCK risk based on the posteriors ##

pPred.all<-c()

yPred.all<-c()

pPred <-c()

yPred <-c()

nr.iter<- length(post.beta0)

nr.datapoints<-length(ket_1.2)

nr.clusters <- length(unique(herds))

post.uj<-matrix(, nrow = nr.iter, ncol = nr.clusters)

# in each iteration and for each herd, draw a value from the normal distribution with mean 0 and sampled posterior standard deviation for the random effects

for (j in 1:nr.clusters) {

uj.hatall <- c()

for (i in 1:nr.iter) {

uj.hat <- rnorm(1, 0, sd=post.sdU[i])

uj.hatall <- c(uj.hatall, uj.hat)

}

post.uj[,j] <-uj.hatall

}

cum.herd <- ave(herd.size, FUN=cumsum)

# predict the risk on SCK for all animals (pPred.all) and use cut-off 0.5 to determine the predicted binary outcomes (yPred.all)

for (d in 1:nr.clusters) {

for (j in (cum.herd[d]-herd.size[d]+1):(cum.herd[d])) {

for (i in 1:nr.iter) {

lp <- post.beta0[i] + post.beta1[i] * parity2[j] + post.beta2[i] * parity3[j] + post.beta3[i] * parity4[j] + post.beta4[i] * spring[j] + post.beta5[i] * winter[j] + post.beta6[i] *summer[j] + post.beta7[i] * bhbz_f[j] + post.beta8[i] * acet_f[j] + post.beta9[i] * ve_rat[j] + post.uj[,d][i]

pPred<- exp(lp)/(1+exp(lp))

pPred.all <- c(pPred.all, pPred)

yPred <- ifelse(pPred<0.5, 0, 1)

yPred.all <-c(yPred.all, yPred)

}

}

}
