# Supplementary material for: Expert opinion as priors for random effects in Bayesian prediction models: Subclinical ketosis in dairy cows as an example
Source: PLoS One. 2021 Jan 14;16(1):e0244752. doi: 10.1371/journal.pone.0244752 (PMC7808599; doi:10.1371/journal.pone.0244752)
Supplement: S3 Appendix — (DOCX) [file pone.0244752.s003.docx]

**Appendix C**

The Instruction

**Background information**

This study is an addition to the earlier study by Van der Drift et al.^^[[1]](#footnote-1)^^ (2012) on subclinical ketosis in Dutch dairy farms. In the original study, data were collected from 123 randomly selected Dutch farms between November 2009 and November 2010. A diagnostic model for subclinical ketosis in early lactation dairy cows (5-60 DIM) in the Netherlands was developed on the basis of the data. The model contained predictors *parity*, *season*, *milk fat-to-protein ratio*, *milk acetone* and *milk* $\beta$*-hydroxybutyrate (BHBA)*. The final analysis was performed with 1,678 cows from 118 farms. For your information, we add the paper (Van der Drift et al., 2012), however, it is not necessary to read the original paper or search for other relevant literature. We would like to ask you to provide your personal opinion based on your existing knowledge and experience.

**This study**

In addition to the animal level predictors, the researcher also recorded feeding management and ration during her visit at the farms. Milk production registration (MPR) summaries at the herd level were obtained from the organization Cattle Improvement Cooperative (CRV). The MPR reports were based on the test day that the researcher visited the farms.

In this study, we would like to include the above mentioned farm level information in the diagnostic model. To do so, we would like to ask you to examine the information from each farm and make your personal opinion about the risk level for subclinical ketosis of the farm relative to other farms. Please note, we are *not* asking for absolute numbers, such as the actual risk or prevalence, but for the position of the farm in the Dutch dairy farm population. We hence assume that you have in your mind an idea of how an ‘average’ farm looks like in the population of the Dutch dairy farms. Then for each farm, we ask you to label the farm as, for instance, average, below average or above average. With below average we mean a lower risk and with above average a higher risk for subclinical ketosis for dairy cows in early lactation. Follow your gut feeling as an expert that knows Dutch dairy farms well.

We provide three different scales and ask you to fill in each scale for each farm. We will first define the three scales and then give two examples.

1. The 2-level scale divides the Dutch dairy farm population into two equal groups. A herd can be placed either to the lower 50% risk group or to the upper 50% risk group of the population (i.e., below or above ‘average’).
2. The 3-level scale divides the population into three equal groups. A herd can be placed either to the lowest 33.3% risk group, or to the middle 33.3% risk group, or to the highest 33.3% risk group of the population.
3. The 5-level scale divides the population into five equal groups. A herd can be placed either to the lowest 20%, or to the highest 20%, or to the three risk groups in between, regarding its position in the population.

As an example, consider that you judge a farm to be of very low risk. On the 2-level and 3-level scale, you will then probably choose the very left box (as illustrated in the picture below). The 5-level scale may be more demanding: is the risk compared to other farms extremely low (first box) or below average but maybe not at the lowest 20% (second box; as illustrated in the picture below). Even when it is difficult to decide between the boxes, please always make one choice. There is no right or wrong answer, what we would like to know is your personal (subjective) guess.

**2-level scale 3-level scale 5-level scale**

50% | 50% 33.3%| 33.3%| 33.3% 20% | 20% | 20% | 20% | 20%


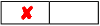

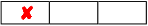

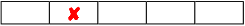


A second example shows the boxes when you consider a farm to be very average. Then the 3-level and 5-level scales are perhaps easy (the middle box; as illustrated in the picture below), but in the 2-level scale you are still asked to make a choice between below average and above average. Give your best guess even if it is difficult to decide.

**2-level scale 3-level scale 5-level scale**

50% | 50% 33.3%| 33.3%| 33.3% 20% | 20% | 20% | 20% | 20%


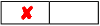

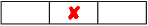

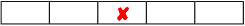


**The scoring form**

The scoring form can be found in the subsequent pages. Each page contains 10 farms (except for the first page), with each row representing one farm. The label in the front indicates the farm ID. For instance, B1 is for farm number one. Please always cross 1 box in each scale for each farm. The 5 farms B1, B2, B3, B4, B32 that were collected but excluded from the final analysis will be used as practice farms. This is presented on the first page, and you may use the 5 farms to familiarize yourself with the three scales. Please contact me^^[[2]](#footnote-2)^^ when you finish the 5 practice farms. We can then make an appointment and I will bring you the materials for the 118 farms.

1. Van der Drift, S. G. A., Jorritsma, R., Schonewille, J. T., Knijn, H. M., Stegeman, J. A. 2012. Routine detection of hyperketonemia in dairy cows using Fourier transform infrared spectroscopy analysis of β-hydroxybutyrate and acetone in milk in combination with test-day information. J Dairy Sci. 95:4886-98. [↑](#footnote-ref-1)
2. Please always feel free to contact me when you have questions or comments.

   Email address: [h.ni@uu.nl](mailto:h.ni@uu.nl) [↑](#footnote-ref-2)
