# Supplementary material for: Expert opinion as priors for random effects in Bayesian prediction models: Subclinical ketosis in dairy cows as an example
Source: PLoS One. 2021 Jan 14;16(1):e0244752. doi: 10.1371/journal.pone.0244752 (PMC7808599; doi:10.1371/journal.pone.0244752)
Supplement: S4 Appendix — (DOCX) [file pone.0244752.s004.docx]

**Appendix D**

**Table 1S**

Frequentist and Bayesian estimates for the regression coefficients and variance of the random effects.

|  | 2012SCK model reproduced  Mean (SE) | | | Bayesian approach  Mean (SE) | |
| --- | --- | --- | --- | --- | --- |
|  |  |  |  |  |  |
| Intercept | -9.097 | 0.940 | -9.538 | | 0.973 |
| Parity 1 | Referent |  | Referent | |  |
| Parity 2 | -0.055 | 0.373 | -0.074 | | 0.407 |
| Parity 3 | 0.690 | 0.348 | 0.699 | | 0.380 |
| Parity >= 4 | 1.362 | 0.313 | 1.391 | | 0.335 |
| Fall | Referent |  | Referent | |  |
| Winter | 0.179 | 0.531 | 0.214 | | 0.566 |
| Spring | 1.514 | 0.520 | 1.621 | | 0.534 |
| Summer | 1.184 | 0.512 | 1.256 | | 0.542 |
| Milk fat-to-protein ratio | 2.534 | 0.535 | 2.677 | | 0.538 |
| Milk acetone (*µ*mol/L) | 0.0100 | 0.002 | 0.0105 | | 0.002 |
| Milk BHBA (*µ*mol/L) | 0.0029 | 0.002 | 0.0029 | | 0.002 |
| Variance of the random effects | 1.792 | 1.339 | 2.230 | | 0.714 |

**
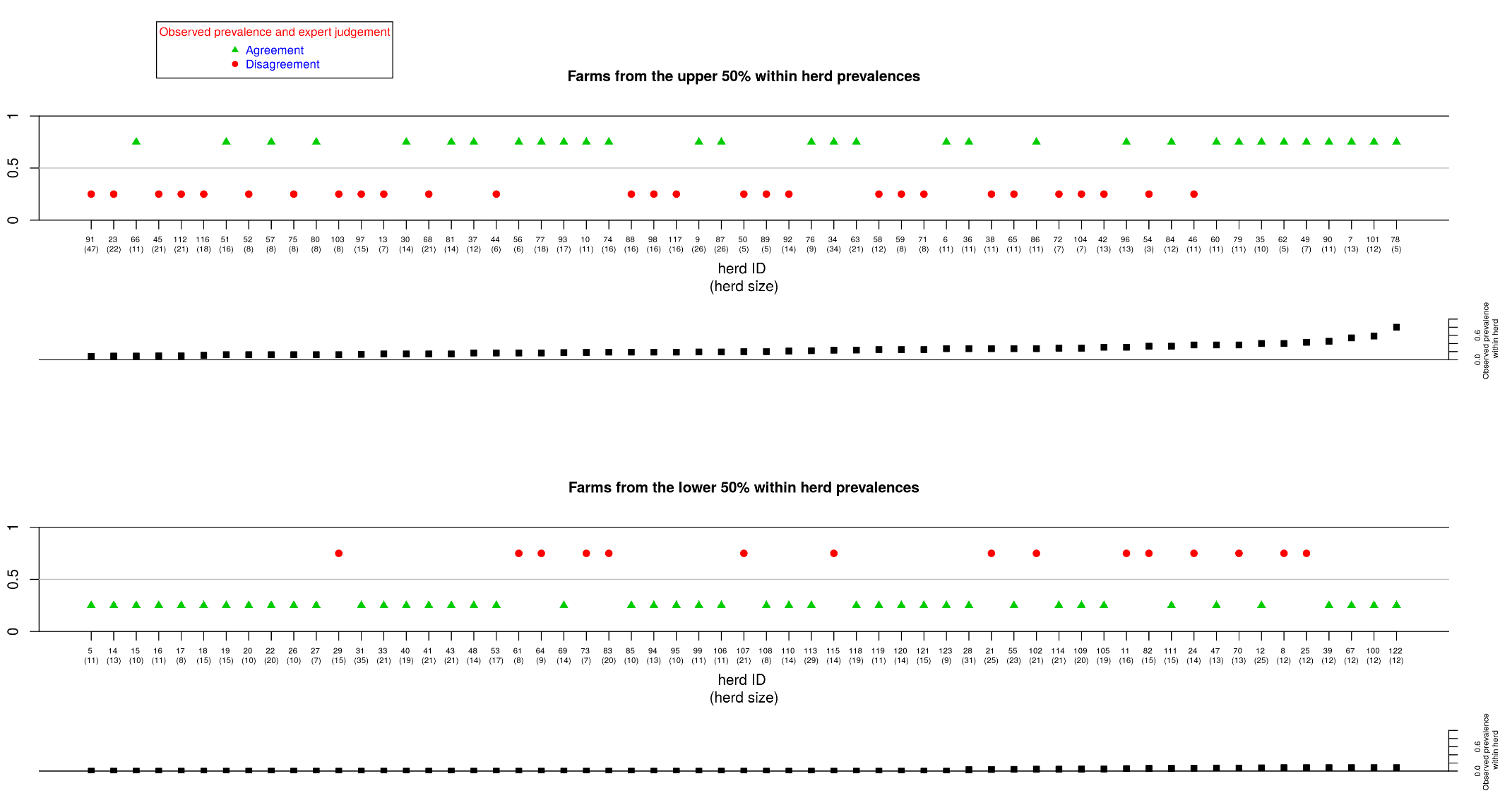
**

**Figure 1S.** The agreement between the observed within herd animal prevalence and the elicited expert opinion in the 2-level scale
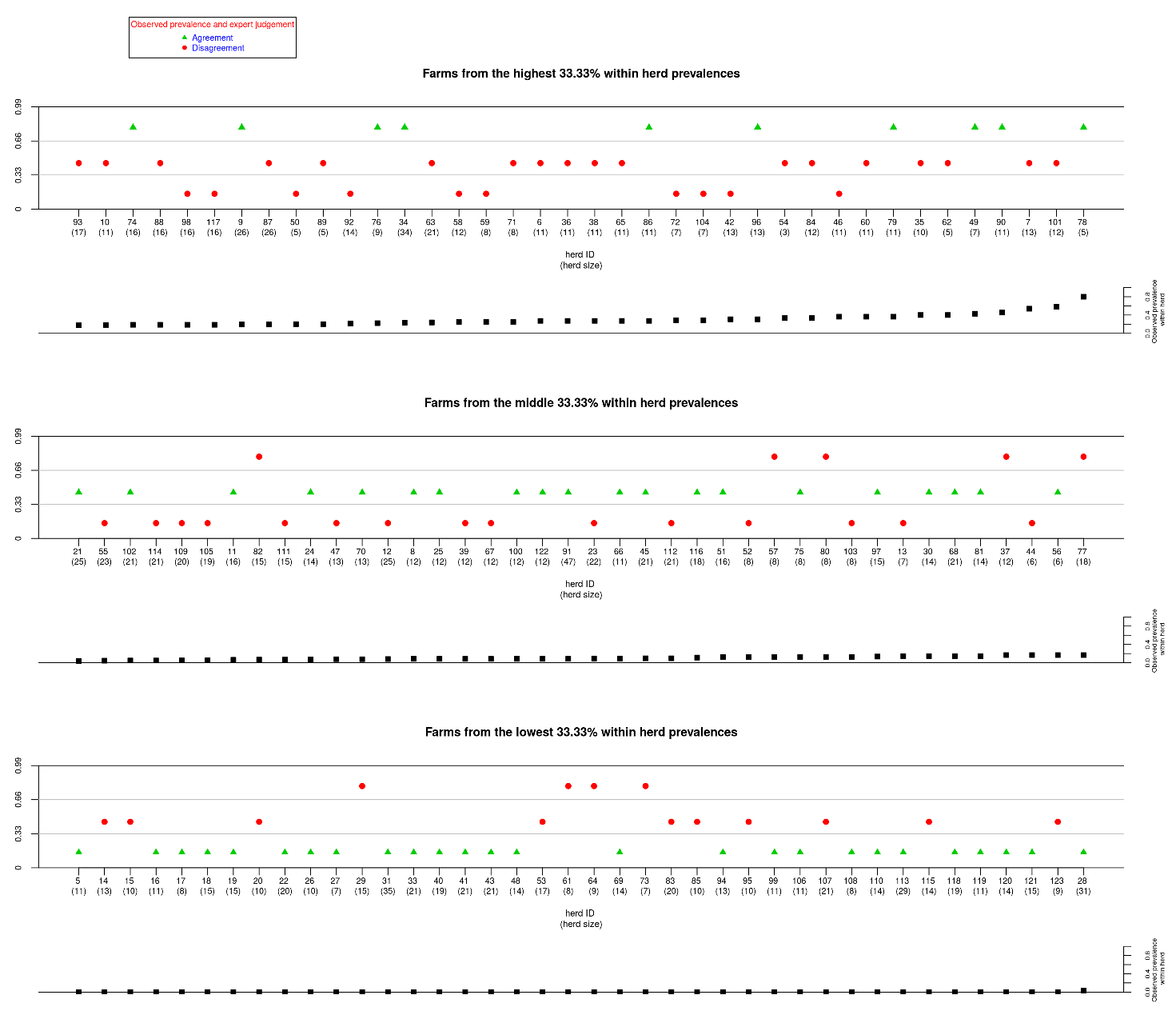


**Figure 2S.** The agreement between the observed within herd animal prevalence and the elicited expert opinion in the 3-level scale


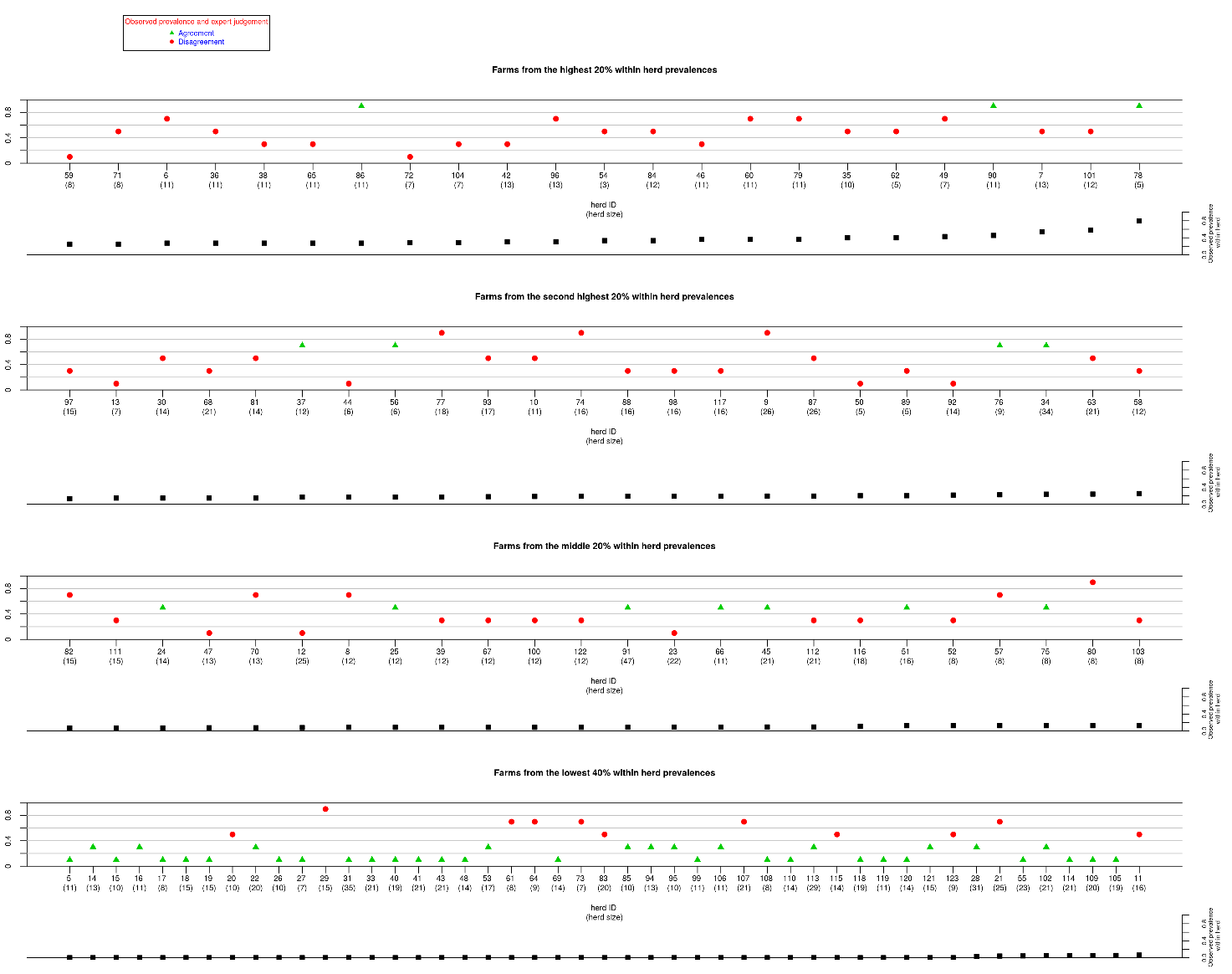


**Figure 3S.** The agreement between the observed within herd animal prevalence and the elicited expert opinion in the 5-level scale

**Table 2S**

The number of correctly/under-/over-estimated within herd animal prevalences (*n* = 118) from the frequentist model and the Bayesian models with herd level prior information incorporated using the optimal cutoff.

|  |  | Correct  Prevalence (%) | Underestimated  Prevalence (%) | Overestimated  Prevalence (%) | Alarm level of prevalence 10% (*n* = 54) | |
| --- | --- | --- | --- | --- | --- | --- |
|  |  |  |  |  | False positives (%) | False negatives (%) |
| FREQ |  | 30 (25.4) | 11 (9.3) | 77 (65.3) | 33 (51.6) | 2 (3.7) |
| Bayes0 |  | 30 (25.4) | 8 (6.8) | 80 (67.8) | 36 (56.3) | 1 (1.9) |
|  |  |  |  |  |  |  |
| Bayes2  (2 levels) | Elicited expert opinion | 44 (37.3) | 20 (16.9) | 54 (45.8) | 19 (29.7) | 9 (16.7) |
|  | Optimal expert opinion | 42 (35.6) | 15 (12.7) | 61 (51.7) | 10 (15.6) | 1 (1.9) |
|  |  |  |  |  |  |  |
| Bayes3  (3 levels) | Elicited expert opinion | 43 (36.5) | 13 (11.0) | 62 (52.5) | 20 (31.2) | 4 (7.4) |
|  | Optimal expert opinion | 48 (40.7) | 12 (10.1) | 58 (49.2) | 13 (20.3) | 5 (9.3) |
|  |  |  |  |  |  |  |
| Bayes5  (5 levels) | Elicited expert opinion | 31 (26.3) | 13 (11.0) | 74 (62.7) | 31 (48.4) | 3 (5.6) |
|  | Optimal expert opinion | 38 (32.2) | 8 (6.8) | 72 (61.0) | 15 (23.4) | 2 (3.7) |

**Table 3S**

Comparison between the Bayesian models with optimal expert opinion under the normal and skew-normal assumption for the random herd effects on animal level measures: area under the curve (AUC), Brier score, calibration slope, sensitivity (Se) and specificity (Sp) using the optimal cutoff, sensitivity using the 95% and 90% specificity cutoffs for the predicted outcomes.

|  |  |  | Optimal expert opinion  (Normal distribution) | | | | Optimal expert opinion  (Skew-normal distribution) | | | |
| --- | --- | --- | --- | --- | --- | --- | --- | --- | --- | --- |
|  | Optimal  value | FREQ | Bayes0 | Bayes2  (2 levels) | Bayes3  (3 levels) | Bayes5  (5 levels) | Bayes0 | Bayes2  (2 levels) | Bayes3  (3 levels) | Bayes5  (5 levels) |
| AUC (%) | 100 | 88.5 | 88.3 | 91.1 | 92.4 | 92.5 | 88.3 | 90.8 | 92.8 | 92.9 |
| Brier score | 0 | 0.069 | 0.069 | 0.062 | 0.059 | 0.058 | 0.070 | 0.069 | 0.064 | 0.060 |
| Calibration slope | 1 | 0.809 | 0.787 | 0.796 | 0.832 | 0.821 | 0.782 | 0.646 | 0.743 | 0.755 |
| Se (optimal cutoff) (%) | 100 | 82.4 | 81.4 | 81.4 | 81.4 | 88.3 | 80.9 | 80.9 | 80.9 | 89.4 |
| Sp (optimal cutoff) (%) | 100 | 83.8 | 83.5 | 85.9 | 86.7 | 80.3 | 84.0 | 86.0 | 87.8 | 80.7 |
| Se (95% Sp cutoff) (%) | 100 | 51.1 | 51.6 | 56.9 | 63.3 | 64.4 | 51.6 | 61.7 | 64.9 | 64.4 |
| Se (90% Sp cutoff) (%) | 100 | 69.7 | 69.1 | 74.5 | 76.1 | 75.0 | 69.1 | 75.0 | 76.6 | 75.5 |

**Table 4S**

The number of correctly/under-/over-estimated within herd animal prevalences (*n* = 118) from the frequentist model and the Bayesian models with optimal expert opinion incorporated and under either normal or skew-normal distribution for the random herd effects using the optimal cutoff.

|  |  | Correct  Prevalence (%) | Underestimated  Prevalence (%) | Overestimated  Prevalence (%) | Alarm level of prevalence 10% (*n* = 54) | |
| --- | --- | --- | --- | --- | --- | --- |
|  |  |  |  |  | False positives (%) | False negatives (%) |
| FREQ |  | 30 (25.4) | 11 (9.3) | 77 (65.3) | 33 (51.6) | 2 (3.7) |
|  |  |  |  |  |  |  |
| Bayes0 | Normal | 30 (25.4) | 8 (6.8) | 80 (67.8) | 36 (56.3) | 1 (1.9) |
|  | Skew-normal | 30 (25.4) | 12 (10.2) | 76 (64.4) | 33 (51.6) | 3 (5.6) |
|  |  |  |  |  |  |  |
| Bayes2  (2 levels) | Normal | 42 (35.6) | 15 (12.7) | 61 (51.7) | 10 (15.6) | 1 (1.9) |
|  | Skew-normal | 49 (41.5) | 19 (16.1) | 50 (42.4) | 5 (7.8) | 1 (1.9) |
|  |  |  |  |  |  |  |
| Bayes3  (3 levels) | Normal | 48 (40.7) | 12 (10.1) | 58 (49.2) | 13 (20.3) | 5 (9.3) |
|  | Skew-normal | 55 (46.6) | 18 (15.3) | 45 (38.1) | 3 (4.7) | 6 (11.1) |
|  |  |  |  |  |  |  |
| Bayes5  (5 levels) | Normal | 38 (32.2) | 8 (6.8) | 72 (61.0) | 15 (23.4) | 2 (3.7) |
|  | Skew-normal | 46 (39.0) | 7 (5.9) | 65 (55.1) | 12 (18.7) | 1 (1.9) |

**Table 5S**

Frequentist and Bayesian parameter estimates based on the training set (94 herds with 1,331 cows).

|  | 2012SCK model reproduced  Mean (SE) | | | Bayesian approach  Mean (SE) | |
| --- | --- | --- | --- | --- | --- |
|  |  |  |  |  |  |
| Intercept | -9.255 | 1.060 | -9.607 | | 1.107 |
| Parity 1 | Referent |  | Referent | |  |
| Parity 2 | -0.055 | 0.427 | -0.028 | | 0.467 |
| Parity 3 | 0.668 | 0.391 | 0.710 | | 0.394 |
| Parity >= 4 | 1.277 | 0.345 | 1.340 | | 0.355 |
| Fall | Referent |  | Referent | |  |
| Winter | 0.046 | 0.588 | 0.057 | | 0.627 |
| Spring | 1.549 | 0.550 | 1.642 | | 0.582 |
| Summer | 1.256 | 0.538 | 1.354 | | 0.589 |
| Milk fat-to-protein ratio | 2.703 | 0.611 | 2.804 | | 0.639 |
| Milk acetone (*µ*mol/L) | 0.0100 | 0.002 | 0.0099 | | 0.002 |
| Milk BHBA (*µ*mol/L) | 0.0028 | 0.002 | 0.0028 | | 0.002 |
| Variance of the random effects | 1.491 | 1.221 | 1.727 | | 0.710 |

**Table 6S**

Animal level measures (*n* = 347*) area under the curve (AUC), Brier score, calibration slope, sensitivity (Se), specificity (Sp) using the optimal cutoff and sensitivity using the 95% and 90% specificity cutoffs for the predicted outcomes.

|  |  |  |  |  | Elicited expert opinion | | | |  |  |
| --- | --- | --- | --- | --- | --- | --- | --- | --- | --- | --- |
|  | Optimal  Value | FREQ | Bayes0 |  |  | Bayes2  (2 levels) | Bayes3  (3 levels) | Bayes5  (5 levels) |  |  |
| AUC (%) | 100 | 83.1 | 83.0 |  |  | 85.7 | 88.1 | 87.5 |  |  |
| Brier score | 0 | 0.082 | 0.082 |  |  | 0.076 | 0.073 | 0.073 |  |  |
| Calibration slope | 1 | 0.719 | 0.701 |  |  | 0.667 | 0.735 | 0.674 |  |  |
| Se (optimal cutoff) (%) | 100 | 72.7 | 72.7 |  |  | 68.2 | 88.6 | 81.8 |  |  |
| Sp (optimal cutoff) (%) | 100 | 85.8 | 83.8 |  |  | 85.8 | 71.3 | 76.6 |  |  |
| Se (95% Sp cutoff) (%) | 100 | 50.0 | 50.0 |  |  | 50.0 | 52.3 | 52.3 |  |  |
| Se (90% Sp cutoff) (%) | 100 | 63.6 | 63.6 |  |  | 63.6 | 61.4 | 65.9 |  |  |

*94 herds with 1,331 cows were randomly selected as the training set, and the remaining 24 herds with 347 cows were subsequently predicted.
