# Supplementary material for: Expert opinion as priors for random effects in Bayesian prediction models: Subclinical ketosis in dairy cows as an example
Source: PLoS One. 2021 Jan 14;16(1):e0244752. doi: 10.1371/journal.pone.0244752 (PMC7808599; doi:10.1371/journal.pone.0244752)
Supplement: Attachment — Submitted filename: Response to Reviewers.docx [file pone.0244752.s006.docx]

We would like to thank the editor and reviewers for providing valuable feedback on our manuscript. Please find below the response to each comment and all the changes we made to the manuscript.

*Editor:*

*Both reviewers report that this is a well-written article and are supportive of its publication. I concur with their view. However, the reviewers have requested for some clarifications in different aspects that would strengthen the manuscript. Please address these comments.*

Thank you for the feedback. We have modified the manuscript as requested.

Reviewer #1:

*The manuscript covered thoroughly the different subject aspects. However, I have raised some concerns and comments, which could improve the quality of the current version of the manuscript.

Title
I think it should be rewritten in a shorter format and consider adding something about Bayesian analysis. I suggest this one “Expert opinion as priors for random effects in a Bayesian prediction model: subclinical ketosis in dairy cows as an example”*

We have modified the manuscript as requested.

*Abstract
It is well written and informative, but I think the authors should add some information about the study population. Similarly, information on the type of the used model is missing. The estimates from the four models should be listed in the abstract.*

We thank the reviewer for the suggestion. Information about the study population is now included in abstract in line 30-33 “Using a dataset of 118 randomly selected Dutch dairy farms participating in a regular milk recording system, the authors proposed a prediction model with milk measures as well as available test-day information as predictors for the diagnosis of subclinical ketosis in dairy cows.”

Further, information on the type of the used model is added to abstract as well in line 35-40 “With the Bayesian prediction modelling approach, we first used non-informative priors for the random effects for model development as well as for prediction. This approach was evaluated by comparing it to the original frequentist model. In addition, herd level expert opinion was elicited from a bovine health specialist using three different scales of precision and incorporated in the prediction as informative priors for the random effects, resulting in three more Bayesian prediction models.”

The estimates from the four models are now listed in line 43-51 “However in this dataset, when elicited expert opinion was incorporated, little improvement was seen at the individual level as well as at the herd level. When the prediction models were applied to the 118 herds, at the individual cow level, with the original frequentist approach we obtained a sensitivity of 82.4% and a specificity of 83.8% at optimal cutoff, while with the three Bayesian models with elicited expert opinion, we obtained sensitivities ranged from 78.7% to 84.6% and specificities ranged from 75.0% to 83.6%. At the herd level, 30 out of 118 within herd prevalences were correctly predicted by the original frequentist approach, and 31 to 44 herds were correctly predicted by the three Bayesian models with elicited expert opinion. ”

*Introduction
The introduction is well-written and addresses the topic satisfactory, however, the authors should give some background on Bayesian modeling and how it works or at least mention the reasons that make it preferable over conventional modeling.*

We thank the reviewer for this suggestion and have added background information on Bayesian modelling in the introduction, line 67-73 “Within the Bayesian framework, a prior distribution for the model parameters reflects the knowledge or uncertainty about the parameters before observing the data. By updating the prior distributions with data, posterior distributions are obtained. One of the advantages of using Bayesian modelling, therefore, is that it can naturally combine multiple sources of evidence [3]. In the study from Ni et al. [2] for instance, (simulated) cluster level expert opinion was used as informative priors for the random effects of new clusters.”

*Line 59. Change “…included in the priors of the random effects …” to “…used as priors of the random effects ...”*

We have modified the manuscript as requested.

*Line 67-69. You need to mention a couple of those attempts (e.g., Krogh et al., 2011, DOI: 10.3168/jds.2010-3816) that worked on the diagnostics of subclinical ketosis.*

*Line 86-88. I think this is more relevant to the introduction. In fact, I encourage the authors to add a short paragraph on bovine ketosis to the section of introduction.*

We agree with the reviewer on this comment and have included references on diagnostics of subclinical ketosis in the introduction, line 81-83 “In dairy science for instance, attempts have been made to develop diagnostic methods to detect subclinical ketosis (SCK) in dairy cows based on routine milk recording data [e.g., 5-6]. “

Furthermore, line 86-88 from the original manuscript in the M&M section is now moved to the introduction (line 83-86) “SCK is considered one of the main metabolic disorders in early lactation dairy cows, which is defined by an increased concentration of ketone bodies in body fluids in absence of clinical signs [7]. Analysis of the concentration of acetone and β-hydroxybutyrate (BHBA) in blood is considered the reference test (e.g., [8]).”

*Line 76-77. Please, explain why you used weakly-informative priors.*

We have changed the word ‘weakly-informative’ to the more conventionally used ‘non-informative’ (e.g., line 36, 95) in veterinary science. A normal distribution with mean 0 and a large variance was used as non-informative priors for the random herd effects. The Bayesian model with non-informative priors for the random effects was applied in order to compare with the original frequentist model from Van der Drift et al. (2012). Further, by including the Bayesian prediction model with non-informative priors for the random effects, it shows the possibility of attaining the random effects in the prediction model even when there is no additional herd level information available.

*Line 77. Using prior information in Bayesian modelling is very common. Priors could be extracted from previous relevant research studies or expert opinions in the field. I think it may deserve to run four models; a model with non-prior information, a model with expert opinions as priors, a model with priors extracted from literature and the last one without priors (zero). With this approach, you can show the variations among the four different model scenarios then compare between them and choose the best one based on DIC (lowest value).*

We agree with the reviewer on using different priors. As it can be seen in the original manuscript, we have included a model without priors/frequentist approach, a model with non-prior information/non-informative prior, and three models with informative priors for the random herd effects using expert opinion captured in 3 different precision scales.

Further, we agree with the reviewer that it is possible and useful to specify informative priors for all parameters in the prediction model on the basis of data and results from literature. However, as the title summarizes, in this paper we focused on investigating the effect of including herd specific information in prediction. When using information extracted from literature, we would not be able to assign herd specific priors, which is the main scientific message of the current paper.

*M&M
Line 94. I think the order of Appendix files should start with A first then B, so on. Here, you are starting with C. do you have a specific reason?*

We have now changed the order of appendices.

Line 90. What do you mean by cow level? This can be understandable for blood but for milk, we have 4 quarters. Please clarify it.

Indeed cows produce milk from 4 quarters. However, in milk recording procedures the milk is sampled from the total milk produced by a cow in a particular milking (sampled from the milking glass, or subsampled from the total milk stream), hence is at cow level. A sample from the next milking is combined with the first sample, to have a morning and evening milking included per cow.

We found this too technical to include in the manuscript, but added in line 127-129 “Milk acetone (97.41$\pm$116.76 μmol/L) and milk BHBA (74.95$\pm$77.82 μmol/L) measures at individual cow level were obtained from routine milk analysis by Fourier transform infrared (FTIR) spectroscopy.”

*Line 92. “.. were randomly selected and visited to collect data..” please, add further details on the method of randomization, what were the selection criteria for inclusion in the study and the exclusion criteria as well. Additionally, what are the type of data that they collected?*

We thank the reviewer for pointing this out. The method of randomization is carefully described in Van der Drift et al (2012). The following text is now added in line 107-110 “In short, a total of 123 Dutch farms were randomly selected from the milk recording organization the Dutch-Flemish Cattle Improvement Cooperative (CRV), which includes 83.8% of all  Dutch  dairy  farmers. The 123 farms were visited on a planned milk recording test day between November 2009 and November 2010 for data collection.”

*Line 95-96. “… prevalence of SCK for the 1,678 cows was 11.2%. …” based on blood samples or milk samples?*

The prevalence of SCK for the 1,678 cows was based on blood samples. This information is now added for clarification in line 115-116 “The overall animal prevalence of SCK for the 1,678 cows was 11.2% based on the reference test results in the blood samples. ”

Line 104. I think it is important to add some descriptive summary statistics about the cow level measures milk acetone, milk BHBA, milk fat-to-protein ratio, parity as well as the herd level measures.

We agree with the reviewer and have included descriptive summary about the cow level as well as herd level measures in line 127-130 “Milk acetone (97.41$\pm$116.76 μmol/L) and milk BHBA (74.95$\pm$77.82 μmol/L) measures at individual cow level were obtained from routine milk analysis by Fourier transform infrared (FTIR) spectroscopy. Milk fat-to-protein ratio (1.33$\pm$0.23), parity of each animal and season during the farm visit at the test day were included as well.”

*Line 107. Clarify the positivity and negativity for the readers. i.e., cow having BHBA threshold of ≥1,200 μmol/L is positive?*

Correct. Cows having BHBA threshold of ≥1,200 μmol/L are considered positive. We have now rephrased the sentence in line 130-132 “Observed binary outcomes were obtained by applying the plasma BHBA threshold of 1,200 µmol/L, above which an animal was considered SCK positive.”

*Line 115. For the Bayesian approach, how did you assessed the convergence of the MCMC chain? did you check the visual inspection of the time-series plots? Did you check the Gelman-Rubin diagnostic plots? How did you assess the significance level in Bayesian analysis?* Please, support your Bayesian analysis with 1-2 references

Information about the convergence assessment is now clarified in the manuscript in line 144-150 “Three Markov chain Monte Carlo (MCMC) posterior chains were sampled. Within each chain, the first 5,000 iterations were discarded as the burn-in phase. The convergence was visually inspected using trace plots. Proper convergence was observed for all chains and the subsequent 20,000 iterations were used for parameter estimates. For the purpose of reducing computational effort in the prediction phase, the 20,000 iterations were thinned by 100, resulting in 200 per chain and 600 in total. Each of the 600 iterations consisted of a sampled value for the regression coefficients and the variance of the random effects respectively.”

*Line 144. I just wonder why the authors decided to consider only the personal opinion of one expert. Why not considering two or three experts and compare between them? Is this expert one of the co-authors of this manuscript? I think the expert should be different from the co-authors for an independent judgment and avoid any possible bias.*

*Line 280-282. That is true. I have raised this issue in my comments. Using more than one expert is more reliable and provides more precise estimates. In addition, it minimizes the mis-classification. Since you have carried out it based on the opinion of one expert thus, you should flag this as a study limitation. On top of that, I strongly suggest to prior information from previous literature (and they are many either at herd or cow level), which could give more robust and precise results.*

We thank the reviewer to mention this point. The expert was independent from the study design and analyses. The expert had no access to cow level data. He was added as co-author because of his contribution on expert elicitation. We agree with the reviewer that in general it is better to consult multiple experts. However, in our dataset, we assumed to know the true within herd prevalence from the blood results for each herd. By using this within herd prevalence information, we created “optimal expert opinion” and checked the maximum improvement the prediction model could gain by adding herd level expert opinion (see line 187-196). Our results show that even with optimal expert opinion, improved cow level prediction remained poor. We deemed it therefore not useful nor ethical to consult multiple experts and asking them to carry out a time-consuming difficult task. For future similar applications where no gold standard test results are available, it is recommended to always consult multiple experts. This is also mentioned in discussion (line 309-322).

Furthermore, as mentioned earlier, one of the main messages of this paper is to include herd specific information as prior for each random herd effect. By using information extracted from literature, we would not be able to assign herd specific priors.

*Line 179-180. Which four Bayesian models?* I think I lost here. I miss the justification for using the weakly-informative priors.

We have rephrased the paragraph in line 204-206 “Given the asymmetric nature of the SCK within herd prevalences, we also investigated the skew-normal distribution with optimal expert opinion for all Bayesian models [17].”

We have further clarified the four Bayesian models in

abstract (line 35-40)

“With the Bayesian prediction modelling approach, we first used non-informative priors for the random effects for model development as well as for prediction. This approach was evaluated by comparing it to the original frequentist model. In addition, herd level expert opinion was elicited from a bovine health specialist using three different scales of precision and incorporated in the prediction as informative priors for the random effects, resulting in three more Bayesian prediction models.”

Introduction (line 94-98)

“Four Bayesian prediction models were investigated. First, a Bayesian prediction model with non-informative priors for the random effects were evaluated by comparing it to the original model. Three more Bayesian models were explored with herd level expert opinion elicited from a bovine health specialist incorporated in the prediction through informative priors for the random herd effects.”

M&M (line 213-216)

“Further, four Bayesian models were specified. We denote the Bayesian prediction model without herd specific information as Bayes0. The Bayesian models with herd level prior information are denoted as Bayes2 (2-level scale), Bayes3 (3-level scale), Bayes5 (5-level scale) respectively.”

Results
*It would be much better if you started the results to briefly describe the study population, cows and herd measures.*

As mentioned in previous answers, this information can now be found

in the introduction section (line 107-110)

“In short, a total of 123 Dutch farms were randomly selected from the milk recording organization the Dutch-Flemish Cattle Improvement Cooperative (CRV), which includes 83.8% of all  Dutch  dairy  farmers. The 123 farms were visited on a planned milk recording test day between November 2009 and November 2010 for data collection.”

and in the M&M section (line 127-132)

“Milk acetone (97.41$\pm$116.76 μmol/L) and milk BHBA (74.95$\pm$77.82 μmol/L) measures at individual cow level were obtained from routine milk analysis by Fourier transform infrared (FTIR) spectroscopy. Milk fat-to-protein ratio (1.33$\pm$0.23), parity of each animal and season during the farm visit at the test day were included as well. Observed binary outcomes were obtained by applying the plasma BHBA threshold of 1,200 µmol/L, above which an animal was considered SCK positive.”

*Line 216. As I mentioned above, it is important to compare between the models using DIC value to choose the best model. you can also use the Youden’s index (Y) to compare the overall performance based on the Se and Sp with the highest value being generally the most preferable at the tested cutoff in table 1.*

*As I mentioned above, I suggest the authors to run 4 models and compare between them using the Deviance Information Criteria (DIC) according to Spiegelhalter et al. (Spiegelhalter, D.J., Best, N.G., Carlin, B.P., van der Linde, A., 2002. Bayesian measures of model complexity and fit (with discussion). J. Roy. Stat.Soc. B 64, 583–640).*

We agree with the reviewer that the DIC is a good criterion for Bayesian model selection. However, model selection is not the goal of this paper. We compare the performance of the different models to describe the effect of adding different amounts of prior information. Theoretically, it is expected that Bayesian models with more precise herd level expert opinion incorporated would provide the best prediction and this was indeed seen in the simulation study from Ni et al. (2018). Here, we explored to what extent improvements were also found in a real data application. Formal model comparison is not part of what we aim to demonstrate.

With respect to model assessment, we agree that Youden’s Index is also useful but we decided to use measures that were the same as in the simulation study, that is the area under the ROC curve (AUC), Brier score (i.e., mean squared error), calibration slope, sensitivity and specificity at the optimal cutoff (defined as maximum sum of sensitivity and specificity). Given the relation/similarity between the last one and Youden’s Index, we decided not to add this criterion to the paper.

*Discussion
Line 291. Another explanation of what exactly? Clarify, please*

We have revised the sentence in line 323-324 “Another explanation for little improvement in prediction both at the individual cow level and at the herd level might be the small sample sized herds in this study.”

*Line 302. “many herds that had zero diseased animals” This is not clear in the manuscript because there is no descriptive statistics were presented or discussed.*

We have rephrased the sentence in line 334-336 “However, 39 out of 118 herds had zero SCK animals which influenced the ICC and the subsequent random effects estimation.”

*I think the discussion still needs further improvements and the authors should focus more on discussing the main findings as well as the interpretation of the difference between the different types of Bayesian models and conventional one. Almost half of the discussion, the authors are discussing the limitations of the study (one expert and small size herds) rather than the main study findings.*

We agree with the reviewer that the main clinical findings of the study should be well addressed. We have now revised part of the first paragraph of the discussion as follows (line 299-305) “However in this dataset, little improvement was seen in prediction resulting from the Bayesian models with elicited expert opinion incorporated in comparison to the prediction from the original frequentist model. The predictive performance from the three Bayesian models with different levels of precision in expert opinion remained poor at the individual cow level. At the herd level, the Bayesian prediction models showed slightly higher diagnostic accuracy, with more within herd prevalences being correctly estimated and less false positives at the alarm level of 10%.”

Next to the clinical conclusions, we consider it even more important to carefully discuss several methodological issues that we encountered when applying our approach to these data. For instance, the inclusion of small sample sized herds reflects the situation within the system in the Netherlands for SCK research. Since this is often seen in observational studies, we feel it is important to discuss challenges and limitations in such scenarios. Further, by eliciting expert opinion from a bovine specialist, we illustrated that it is in practice applicable to add herd level expert opinion to prediction. The fact that we used just one expert is a limitation (though adding more would not have been helpful and ethical in this study) that needs to be addressed for potential future clinical applications.

*The conclusion is very generic and does not precisely reflect the study. The authors developed a Bayesian model and compared it with a conventional model for subclinical ketosis in cattle. This is not clear in your conclusion. I like the way you wrote the conclusion in the abstract. It clearly reflects and summarize the main point from this research study.*

We thank the reviewer to point this out and have revised the conclusion (line 352-358) “This study illustrates how the Bayesian prediction modelling approach can take the clustering effect into account and how cluster level expert opinion can be combined with individual level data. However in this dataset, incorporation of elicited expert opinion did not improve prediction at the individual level nor at the herd level. Therefore, further investigation of the potential gain of using this approach requires applications in studies where the between cluster variance is relatively large and where all clusters harbor individuals with the outcome under study.”

*Where is the conflict of interest statement?*

The conflict of interest statement is now added to the manuscript “All authors state that they have no conflict of interest.” (line 386-387).

*Reviewer #2: This is quite a nice piece of work that I enjoyed reading. Well done to the authors.*
*I just have a few general comments and a small number of specific ones. There is a significant problem of overfitting when using training data to evaluate the predictive ability of a model. Conventionally, datasets are split into training and testing datasets to avoid this. The authors should consider this approach as it may change the results of their study. In addition to this, it is not clear when reading the abstract which data are being predicted. It is important to be transparent about whether these predictions are applied to new (test) data, or the same data that the model was built on.*

We thank the reviewer for pointing this out and we agree that splitting data in a training set and a test set for prediction modelling is the preferred approach in an empirical study. The main reason that we used the same dataset for training as well as for test was that in the original study from Van der Drift et al. (2012), the authors also used the same data for model development as well as for prediction. In order to objectively compare the predictive performance between the original frequentist model and the Bayesian models, we therefore took the same approach. After rethinking this approach, given that the main message of the paper is the inclusion of informative priors for the random effects, we feel that an additional analysis based on training and test set would merely add complexity to the paper. Hence we prefer to keep the analysis and comparisons as it is.

We have now revised the abstract to make it clear that the same dataset was used in this study for model development as well as for prediction (line 45-51) “When the prediction models were applied to the 118 herds, at the individual cow level, with the original frequentist approach we obtained a sensitivity of 82.4% and a specificity of 83.8% at the optimal cutoff, while with the three Bayesian models with elicited expert opinion, we obtained sensitivities ranged from 78.7% to 84.6% and specificities ranged from 75.0% to 83.6%. At the herd level, 30 out of 118 within herd prevalences were correctly predicted by the original frequentist approach, and 31 to 44 herds were correctly predicted by the three Bayesian models with elicited expert opinion.”

*Line 76-79 - reads like a summary of the materials and methods - don't think this is required here*

This part is now removed.

*Line 86-89 reads more like introductory material*

We agree with the reviewer and have moved this part to the introduction, line 81-86.

*Line 94-97 - What were the criteria for sampling per farm? (why were only 3 sampled one farm?)
What stage of lactation were animals sampled in?*

The following text is now added in line 110-112 for clarification “On each farm, all cows between 5 and 60 days in milk (DIM) were blood sampled, which is the risk group for SCK. As a consequence, there were not many eligible cows present on smaller farms.”

*Line 148-150 - Was this information also used in the predictive model, or was it only available to the expert? They are herd level data so probably just the expert but it should be made clear.*

The collected herd level feeding management and milk recording documents during the farm visit was not used in the predictive model as herd level variables, but only given to the expert as a proxy for being at the farm. We have rephrased this part in line 172-176 “As previous research indicated that the risk for SCK is related to routine feeding practices (e.g., [10]) and herd level milk yield (e.g., [11]), herd level feeding management and herd level milk production documents that were collected during the farm visit [9] were provided to the expert as a proxy for a farm visit. This herd level information on feeding and milk production was hence incorporated in the prediction model through elicited expert opinion.”

*Line 225 - Can you expand on the herd level predictions? I think you probably need some additional information on this on the materials and methods also. In lines 225-233, It appears that you are predicting the probability for each cow, and then converting this to a predicted App Prev for each herd. But you will need a cut off point above which an animal will be considered predicted positive? The numbers from 228-233 read more like the individual level rather than the herd? Can you clarify this please?*

Correct. The predicted within herd prevalences were based on the predicted binary value of each cow. We have now rephrased the paragraph in line 252-256 “Predicted within herd animal prevalences were compared to the observed prevalences based on the reference test results in blood samples. The predicted prevalence of each herd was calculated from the predicted binary outcome (with disease probability >0.50 as positive) from each cow within the herd using the optimal cutoff (defined as maximum sum of sensitivity and specificity).”
