# Supplementary material for: Expert opinion as priors for random effects in Bayesian prediction models: Subclinical ketosis in dairy cows as an example
Source: PLoS One. 2021 Jan 14;16(1):e0244752. doi: 10.1371/journal.pone.0244752 (PMC7808599; doi:10.1371/journal.pone.0244752)
Supplement: Attachment — Submitted filename: Response to Reviewers.docx [file pone.0244752.s007.docx]

We would like to thank the editor and reviewers for providing valuable feedback on our manuscript. Please find below the response to each comment and all the changes we made to the manuscript.

Editor:

Thank you for submitting your manuscript to PLOS ONE. After careful consideration, we feel that it has merit but does not fully meet PLOS ONE’s publication criteria as it currently stands. Therefore, we invite you to submit a revised version of the manuscript that addresses the points raised during the review process.

Thank you for your revised manuscript and pro-actively addressing the reviewers' suggestions. Both reviewers are supportive of the manuscript being accepted, provided their additional suggestions are included. I concur with their view. Particularly, regarding the additional discussion points raised by reviewer #2, which are essential.

Thank you for the feedback. We have modified the manuscript as requested.

Reviewer #1:

Thank you for addressing the raised comments appropriately and implementing the necessary modifications within the manuscript. Your responses are satisfactory and very thorough. The quality of the new version of the manuscript looks much better and more consistent. I do not have further comments except just a minor comment. I suggest adding the R script of the Bayesian model preferably in a text format as a supplementary file/ Appendix.

We thank the reviewer for the feedback. We have added the R script of the Bayesian model in a text format in Appendix B.

Line 157-159 in the manuscript also informs this addition “The R code for the Bayesian prediction model without the incorporation of expert prior knowledge can be found in Appendix B.”

Reviewer #2:

The authors have done a good job of addressing the comments of both reviewers. I just have two follow up general comments:

I think the authors still need to discuss the impact of using the same data to build the model to evaluate its predictive ability. Surely the reason such analyses are undertaken is that they could be of some practical use in the field? In that regard, there is no point in developing a model that can predict very well within the dataset it is constructed on but falls over when applied to new data. Ultimately it us up to the reviewers how they address this, but at the very least this should be clearly and transparently discussed in the discussion section. I do not think that just because the previous authors did it this way is a good explanation for the approach taken.

We thank the reviewer for pointing this out and we agree with the reviewer that it should be transparently discussed in the paper. We have rerun the Bayesian models with 80% of the herds randomly selected as the training set (94 herds with 1,331 cows) and the remaining 20% herds as test set (24 herd with 347 cows).

We have added this information under an extra subsection in results, line 295-304

“Splitting data into a training and a test set

In order to compare objectively between results from the frequentist model in the original study Van der Drift et al. (2012) and the Bayesian prediction models in this study, we used the same dataset for model development as well as for model prediction as it was done in the original study. However, we additionally performed model comparisons based on a 80/20 training/test set approach as follows. About 80% of the 118 herds were randomly selected for model development, resulting in 94 herds with 1,331 cows. The number of cows per herd was approximately 14 on average and 32 out of the 94 herds had zero diseased animals. The parameter estimates from the frequentist as well as the Bayesian approach of the training set are shown in Table 5S, while the prediction results on the test set are found in Table 6S.”

And in discussion line 363-367

“The regression models based on the training set showed very similar parameter estimates to the full dataset, albeit with larger standard errors for the regression coefficients and lower variance for the random effects. The model assessments on the test set showed less favorable prediction results for all methods, as was to be expected, but did not alter our conclusions about the comparisons between the methods.”

Regarding sampling criteria, the authors recruited cows between 5-60 DIM. However, within this range, two different subtypes of SCK occur, that is Type I in the latter half of the window and Type II SCK in the earlier part of the window. Given these subtypes have two different physiological mechanisms, it is reasonable to assume that the risk factors for each subtype might be different. How have the authors dealt with this?

We thank the reviewer for the feedback. We acknowledge that there are theories on two slightly different pathogenesis of ketosis in cows. The reported prevalence of the disease (Van der Drift et al., 2012) does not support a clear distinction in the two types and it is to our knowledge not used in clinical practice. We have therefore not explicitly addressed this issue, but assume that the included risk factors will cover both physiological subtypes of ketosis.
